# Unusual presentation of a severely ill patient having severe fever with thrombocytopenia syndrome: a case report

**DOI:** 10.1186/s13256-016-1192-0

**Published:** 2017-02-03

**Authors:** Masahiko Kaneko, Masaki Maruta, Hisaharu Shikata, Kengo Asou, Hiroto Shinomiya, Tadaki Suzuki, Hideki Hasegawa, Masayuki Shimojima, Masayuki Saijo

**Affiliations:** 1 Department of Internal Medicine, Uwajima City Hospital, 1-1 Goten-machi, Uwajima City, Ehime 798-8510 Japan; 2 Department of Neurosurgery, Ehime University Graduate School of Medicine, Shitsukawa, Toon City, Ehime 791-0295 Japan; 3 Ehime Prefectural Institute of Public Health and Environmental Science, 8-234 Sanbancho, Matsuyama, Ehime 790-0003 Japan; 4 Department of Pathology, National Institute of Infection Diseases, 1-23-1 Toyama, Shinjuku-ku, Tokyo, 162-8640 Japan; 5 Department of Virology 1, National Institute of Infectious Diseases, 4-7-1 Gakuen, Musashimurayama, Tokyo, 208-0011 Japan

**Keywords:** Severe fever with thrombocytopenia syndrome, Encephalitis/encephalopathy, Reversible splenial lesion syndrome, Serum viral load, Case report

## Abstract

**Background:**

Severe fever with thrombocytopenia syndrome is an emerging infectious disease caused by a novel phlebovirus belonging to the family Bunyaviridate. Emergence of encephalitis/encephalopathy during severe fever with thrombocytopenia syndrome progression has been identified as a major risk factor associated with a poor prognosis. Here we report the case of a severely ill patient with severe fever with thrombocytopenia syndrome virus-associated encephalitis/encephalopathy characterized by a lesion of the splenium, which resolved later.

**Case presentation:**

A 56-year-old Japanese man presented with fever and diarrhea, followed by dysarthria. Diffusion-weighted magnetic resonance imaging demonstrated high signal intensity in the splenium of the corpus callosum. The severe fever with thrombocytopenia syndrome virus genome was detected in our patient’s serum, and the clinical course was characterized by convulsion, stupor, and hemorrhagic manifestations, with disseminated intravascular coagulation and hemophagocytic lymphohistiocytosis. Supportive therapy not including administration of corticosteroids led to gradual improvement of the clinical and laboratory findings, and magnetic resonance imaging demonstrated resolution of the splenial lesion. The serum severe fever with thrombocytopenia syndrome viral copy number, which was determined with the quantitative reverse-transcription polymerase chain reaction, rapidly decreased despite the severe clinical course. Our patient’s overall condition improved, allowing him to be eventually discharged.

**Conclusions:**

Patients with encephalitis/encephalopathy due to severe fever with thrombocytopenia syndrome virus infection may have a favorable outcome, even if they exhibit splenial lesions and a severe clinical course; monitoring the serum viral load may be of value for prediction of outcome and potentially enables the avoidance of corticosteroids to intentionally cause opportunistic infection.

## Background

Severe fever with thrombocytopenia syndrome (SFTS) is a recently identified infectious disease endemic to China, South Korea, and Japan, with a reported fatality rate between 12% and 30% [1]. The emergence of central nervous system (CNS) manifestations during SFTS progression has been identified as a major risk factor for mortality [2].

The presence of reversible lesions that involve the splenium of the corpus callosum (SCC) has been reported in patients with a broad spectrum of diseases and conditions and is referred to as reversible splenial lesion syndrome (RESLES) [3]. The magnetic resonance imaging (MRI) features of RESLES include reversible lesions limited to the splenium of the corpus callosum (SCC) or to the SCC and frontal white matter; hyperintense signals on T2-weighted images (T2WI), fluid-attenuated inversion recovery (FLAIR) images and diffusion-weighted images (DWI); with low apparent diffusion coefficient (ADC) values and hypo- or iso-intense signals on T1-weighted imaging (T1WI) sequences with no contrast enhancement [4, 5].

The exact pathophysiology and the specific site predilection of transient SCC lesions in RESLES are not understood. It has been suggested that transient SCC lesions likely reflect rapidly resolving intramyelinic edema or the influx of inflammatory cells and macromolecules, combined with related cytotoxic edema and hypotonic hyponatremia, which result from infection [6–8].

Here we report the case of a severely ill patient with SFTS virus-associated encephalitis/encephalopathy in whom follow-up magnetic resonance imaging (MRI) demonstrated a lesion in the SCC. This lesion eventually resolved, and our patient made a complete clinical recovery within a few weeks. Analysis of the SFTS viral load in our patient’s serum during hospitalization demonstrated a rapid decrease.

## Case presentation

A 56-year-old Japanese man, who had been engaged in agricultural activities in a hilly rural area of Japan, presented to a local hospital in August, with a 2-day history of fever and diarrhea. Dysarthria and irritability were also evident. He had long-standing diabetes mellitus that had been well controlled with metformin. His vital signs were normal except for pyrexia (temperature of 38.5 °C).

**Fig. 1:**
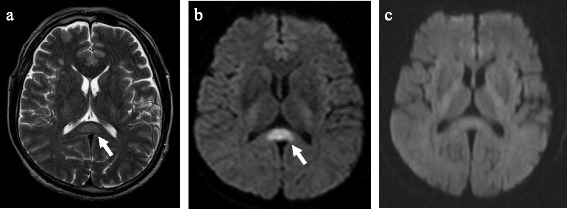
T2- and diffusion-weighted images. T2-weighted (**a**) and diffusion-weighted (**b**) brain magnetic resonance images on the 2^nd^ day showing high-intensity lesions in the splenium of the corpus callosum (*arrow*). The splenial lesions had completely disappeared on the 5^th^ day (**c**)

Laboratory tests demonstrated leukopenia, thrombocytopenia, hyponatremia, and increased levels of aspartate aminotransferase (AST), alanine aminotransferase (ALT), lactate dehydrogenase (LDH), and creatine kinase (CK). Brain MRI revealed a hyperintense lesion in the SCC on T2- (Fig. 1a) and diffusion-weighted (DW) (Fig. 1b) image sequences. A preliminary diagnosis of cerebral infarction was made, and intravenous administration of edaravone was started. However, his clinical condition had not improved by the 3^rd^ day after illness onset, and he was, therefore, transferred to our hospital.

On admission, our patient’s Glasgow score (GCS) was 12 (E3, V3, M6), and he showed limb tremor and scanning speech. A tick bite wound was observed on the anterior aspect of his right ankle.

Blood tests showed leukopenia with a shift to the left, 2.8 × 10^9^/L (normal range: 4.0–9.0) with 48% bands and 1% of atypical lymphocyte.

The other laboratory tests yielded the following results: platelet count, 59 × 10^9^/L (150–450); and hemoglobin, 14.7 g/dL (12–16); C-reactive protein, 1.51 mg/dL (0–0.3); AST, 159 U/L (13–33); ALT, 59 U/L (8–42); LDH, 429 U/L (100–200); creatinine, 1.21 mg/dL (0.36–1.06); CK, 2559 U/L (62–287); serum ferritin, 1980 U/mL (122–496); and sodium levels, 129 mmol/L (135–149).

**Fig. 2:**
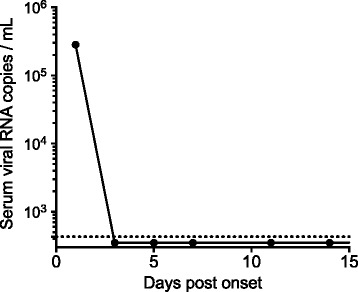
Change in the severe fever with thrombocytopenia syndrome viral copy numbers in the serum of the present patient. The viral copy number on the 1^st^ day after the onset of illness was 2.8 × 10^5^ copies/mL and then rapidly decreased during the remaining period of hospitalization. The *horizontal dashed line* indicates the median detection limit for the quantitative reverse-transcription polymerase chain reaction assays (0.43 × 10^3^ copies/mL). RNA, ribonucleic acid

Contrast-enhanced whole-body computed tomography revealed slight enlargement of the mediastinal, axillary, and inguinal lymph nodes. On the 5^th^ day of illness, reverse-transcription polymerase chain reaction (RT-PCR) for SFTS virus (SFTSV) using a blood sample had a positive result, confirming that our patient had SFTSV infection (Fig. 2).

**Fig. 3:**
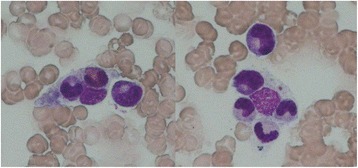
Microscopic findings under May-Giemsa staining. Hemophagocytosis is present in the bone marrow (×1000)

The laboratory values on that day were as follows: platelet count, 31 × 10^9^/L; fibrin/fibrinogen degradation products (FDP), 49.0 μg/mL (0–1); soluble CD25, 2402 U/mL: and increases in the AST, LDH, CK, and serum ferritin levels to 159 U/L, 429 U/L, 4016 U/L, and 24667 U/mL, respectively. A bone marrow smear showed mild hemophagocytosis (Fig. 3).

These laboratory findings fulfilled the diagnostic criteria for acute disseminated intravascular coagulation (DIC) [9] and the clinical diagnosis of secondary hemophagocytic lymphohistiocytosis (HLH) was made by the revised diagnostic criteria including fever, bicytopenia, hepatitis, high level of serum ferritin and sIL-2R, and hemophagocytosis in bone marrow [10].

Thereafter, our patient’s CNS manifestations deteriorated, and he exhibited convulsion and stupor as well as hemorrhagic manifestations, such as gingival bleeding and melena. Lumbar puncture was not performed due to DIC. However, a follow-up DW brain MRI scan on the 5^th^ day of illness demonstrated complete disappearance of the SCC signal enhancement (Fig. 1c).

Laboratory findings indicated gradual improvement after the 6^th^ day of illness. Moreover, hemorrhagic manifestations disappeared on the 9^th^ day of illness, and the CNS disturbance was resolved by the 16^th^ day. Our patient was discharged on the 24^th^ day after illness onset with an overall improvement in his condition. We treated our patient with symptomatic and supportive measures. Throughout the course, our patient neither required intensive care nor was treated with the administration of corticosteroids. He was treated with fluid replacement for dehydration and platelet transfusion. The clinical findings are summarized in Table 1.

**Table 1:** Laboratory findings of the presented case of severe fever with thrombocytopenia syndrome (SFTS)

Laboratory findings	Reference range	Days after illness onset						
		2	3	5	6	9	15	19
WBC (×10^9^/L)	4.0–9.0	1.4	2.8	2.63	3.19	4.36	5.95	6
Hemoglobin (g/dL)	12–16 (men)	15.3	14.7	16.4	16.4	11.4	11.6	10.7
Platelets (×10^9^/L)	150–450	86	59	31	17	30	205	342
AST (U/L)	13–33	145	159	628	362	131	40	28
ALT (U/L)	8–42	55	59	125	93	96	44	27
LDH (U/L)	100–200	360	429	1611	1128	472	326	189
Amylase (U/L)	33–120	NA	116	279	314	179	107	107
Lipase (U/L)	13–49	NA	NA	421	896	297	117	117
CRP (mg/dL)	0–0.3	NA	1.51	6.09	NA	NA	1.21	NA
Creatinine (mg/dL)	0.36–1.06	1.29	1.21	1.35	1.14	0.69	0.71	0.71
CK (U/L)	62–287	2276	2559	4016	4912	699	113	65
CK-MB (U/L)	0–24	NA	37	69	NA	NA	NA	NA
Ferritin (U/mL)	122–496	NA	1980	24667	NA	2209	413	413
Sodium (mmol/L)	135–149	125	129	137	143	137	NA	140
PT/INR	0.8–1.3	1.09	0.95	1.08	NA	NA	NA	0.91
aPTT (s)	24–36	59.1	40.9	100	NA	NA	32.6	32.6
FDP (μg/mL)	0–5	NA	NA	40.5	NA	2.5	NA	3.1
D-dimer (μg/mL)	0–1	NA	2.9	15.6	NA	NA	NA	2.3
Urine protein (mg/day)		684	NA	3845	3260	53163	813	685

*NA* not available, *WBC* white blood cells, *AST* aspartate aminotransferase, *ALT* alanine aminotransferase, *LDH* lactate dehydrogenase, *CRP* C-reactive protein, *CK* creatine kinase, *PT/INR* prothrombin time/international normalized ratio, *aPTT* activated partial thromboplastin time, *FDP* fibrin/fibrinogen degradation products

The SFTS viral load in serum samples collected from our patient, on days 1, 3, 5, 7, 11, and 14 after illness onset were determined by quantitative RT-PCR (qRT-PCR), as described previously [11, 12]. On admission, there had been 2.8 × 10^5^ copies/mL, but the viral load rapidly decreased during the remaining period of hospitalization (Fig. 2).

## Discussion

We have reported the case of a severely ill patient with SFTSV-associated encephalitis/encephalopathy in whom an SCC lesion was detected, but showed spontaneous resolution. The presence of a reversible SCC lesion has been reported in patients with a broad spectrum of diseases and conditions, and is referred to as RESLES [3]. Infection is the most common cause of this abnormality. To the best of our knowledge, this is the first reported case of RESLES in a patient with SFTSV infection.

The common neurological symptoms of RESLES associated with encephalitis/encephalopathy may include delirium, short-term disturbance of consciousness, and seizures, but usually patients show complete recovery without neurological sequelae after a short disease course [13, 14]. Although CNS manifestations are common in patients with SFTS, signs of severe CNS disturbance, such as coma and convulsion, are thought to be major clinical indicators of poor prognosis [2]. An epidemiologic study of 538 patients with SFTS revealed that 19.1% of them developed encephalitis and 44.7% of the 19.1% died [15].

In the present case, it was not clear whether the severe CNS disturbance was due to the severe clinical course of SFTS itself or the SCC lesion that developed secondarily. Achalia *et al*. speculated that the prognosis of RESLES may depend on the underlying disorder and not on other factors [16]. The worsening CNS manifestations, hemorrhagic manifestations, and laboratory data in the present patient were consistent with severe SFTSV infection. HLH may also have contributed to disease severity and clinical outcome [17]. HLH results from excessive activation of the immune system, leading to uncontrolled cytokine release. Although the mechanism responsible for reversible SCC lesions is unknown, Tada *et al*. postulated that intra-myelinic edema or an inflammatory infiltrate might account for the transient decrease in the ADC value [4]. In the present case, we suspect that an inflammatory infiltrate caused by cytokine cascades under conditions of HLH may have been involved in the pathogenesis of the reversible SCC lesion.

SFTS patients with severe complications such as secondary HLH often receive corticosteroids, which repress immune functions. SFTS patients are somewhat immunodeficient [18] or have damaged immune systems with low levels of CD3+ and CD4+ T lymphocytes [19, 20], which may be due to a viral-associated hemophagocytic syndrome. Such immunodeficiency has an important role in disease progression, disease severity, and clinical outcome. Therefore, the administration of corticosteroids for the treatment of HLH, is controversial, because it sometimes worsens the infectious disease status including causing opportunistic infections. In fact, some lethal SFTS cases have involved patients with a history of early treatment with dexamethasone. In particular, two autopsy cases of Japanese SFTS patients, who received corticosteroids for hemophagocytosis, complicated by *Aspergillus* or *Mucor* infection were reported [21]. It is noteworthy that in the patient with *Aspergillus* infection, the thrombocytopenia and other organ pathologies were improving, although the aggressive pulmonary aspergillosis exacerbated the clinical course and resulted in death in the patient. Because the use of corticosteroids in severely ill SFTS patients may be possible to induce opportunistic infections, a prospective study is needed to evaluate the efficacy of corticosteroid therapy.

It appears that SFTS viral load is related to morbidity [12]. It has been reported that the SFTS viral load in acute-phase peripheral blood samples was significantly higher in patients who died than in those who survived, and that a high titer of ≥10^8^ copies/mL led to fatal outcomes [18, 22]. The mean viral load in the survivor group was 10^5^ copies/mL, which were consistent with the findings reported by Zhang *et al.* [18]. Thus, there is a possibility that in this case report the serum SFTS viral load elevation on admission we see, which was equivalent to that in the survivors reported by Zhang *et al.*, contributed to the favorable outcome, even though the patient was severely ill. Therefore, a rapid decline in the viral load revealed by periodic monitoring may be useful in predicting the outcome in severely ill patients with SFTS.

## Conclusions

The present case suggests that SFTSV infection can trigger reversible SCC lesions. Even if patients show unexpectedly severe and prolonged CNS disturbance during SFTS progression, monitoring of the serum SFTS viral load in the acute phase may reveal a rapid decline that is predictive of a favorable outcome and may enable the avoidance of the use of corticosteroids, which potentially cause opportunistic infection.
